# Prevalence of cardiovascular-kidney-metabolic syndrome in Korea: Korea National Health and Nutrition Examination Survey 2011-2021

**DOI:** 10.4178/epih.e2025005

**Published:** 2025-02-14

**Authors:** Sung-Bin Hong, Ji-Eun Kim, Seung Seok Han, Joseph J. Shearer, Jungnam Joo, Ji-Yeob Choi, Véronique L. Roger

**Affiliations:** 1 Department of Biology Education, Seoul National University, Seoul, Korea; 2 Heart Disease Phenomics Laboratory, Epidemiology and Community Health Branch, National Heart, Lung, and Blood Institute, National Institutes of Health, Bethesda, MD, USA; 3 Institute of Health Policy and Management, Seoul National University Medical Research Center, Seoul, Korea; 4 Division of Nephrology, Department of Internal Medicine, Seoul National University Hospital, Seoul National University College of Medicine Seoul, Korea; 5 Office of Biostatistics Research, National Heart, Lung, and Blood Institute, National Institutes of Health, Bethesda, MD, USA; 6 Department of Biomedical Sciences, Seoul National University Graduate School, Seoul, Korea; 7 BK21Plus Biomedical Science Project, Seoul National University College of Medicine, Seoul, Korea; 8 Institute of Health Policy and Management, Seoul National University Medical Research Center, Seoul, Korea; 9 Cancer Research Institute, Seoul National University, Seoul, Korea

**Keywords:** Cardiovascular diseases, Chronic kidney diseases, Metabolic syndrome, Socioeconomic status, Pandemic

## Abstract

**OBJECTIVES:**

The American Heart Association (AHA) recently defined cardiovascular-kidney-metabolic (CKM) syndrome to better characterize the associations among cardiovascular, kidney, and metabolic diseases. Although about 9 in 10 United States adults have at least 1 risk factor for CKM syndrome, its prevalence in other populations is less understood. To fill this gap, we examined the prevalence of CKM syndrome in Korea and its association with demographic and socioeconomic status (SES).

**METHODS:**

Using data from the Korean National Health and Nutrition Examination Survey between 2011 and 2021, we calculated the prevalence of CKM syndrome across the following stages: stage 0 (no risk factors), stage 1 (excess or dysfunctional adiposity), stage 2 (other metabolic risk factors or chronic kidney disease), and stages 3-4 (subclinical/clinical cardiovascular diseases) among adults aged ≥20 years. Weighted analyses were used to estimate prevalence and 95% confidence intervals (CIs) for each CKM syndrome stage, stratified by age, gender, and SES factors.

**RESULTS:**

Among 54,994 Korean adults, the prevalence of CKM syndrome was as follows: stage 0 (25.2%; 95% CI, 24.7 to 25.8), stage 1 (19.3%; 95% CI, 18.9 to 19.7), stage 2 (51.6%; 95% CI, 51.1 to 52.2), and stages 3-4 (3.9%; 95% CI, 3.7 to 4.0). The prevalence of stages 2 and 3-4 was higher in men than in women. In addition, stages 3-4 were more prevalent among rural residents and those with lower education or income.

**CONCLUSIONS:**

About 3 out of 4 Koreans are at risk for CKM syndrome. These findings highlight that CKM syndrome is a global health problem and that interventions are urgently needed to prevent further progression.

## GRAPHICAL ABSTRACT

[Fig f6-epih-47-e2025005]

## Key Message

Recently, the need for an integrated approach to managing cardiovascular-kidney-metabolic (CKM) syndrome has been emphasized. This study found that 74.8% of Korean adults aged 20 and older had a risk for CKM syndrome. Moreover, the prevalence is increasing, highlighting the necessity of proper management.

## INTRODUCTION

The American Heart Association (AHA) recommends defining cardiovascular disease (CVD), chronic kidney disease (CKD), and metabolic syndromes collectively as cardiovascular-kidney-metabolic (CKM) syndrome [1]. CKM syndrome refers to a systemic condition characterized by metabolic risk factors, CKD, and CVD, which together result in multi-organ dysfunction and an elevated risk of adverse cardiovascular events and mortality [2]. According to the Global Burden of Disease Study 2021, diseases related to CKM syndrome—including CVD—are among the leading causes of global deaths [3]. In addition, CKM syndrome can affect almost every major organ system, contributing to kidney failure and cancer, which pose significant clinical challenges [4,5]. Some studies have investigated the complex interrelationships among these conditions [6-11]. Although research on individual components of CKM syndrome and the associations between metabolic diseases and CVD has been conducted in Korea [12-17], the overall prevalence of CKM syndrome in Korea has not been examined. Therefore, understanding the progressive pathology of CKM syndrome is critical for preventing CVD morbidity and mortality, rather than focusing solely on each individual condition.

The current study aimed to investigate the prevalence and annual trends of CKM syndrome stages from 2011 to 2021 using data from the Korean National Health and Nutrition Examination Survey (KNHANES). Additionally, we examined the association between socioeconomic status (SES) and CKM syndrome, assessing changes during the coronavirus disease 2019 (COVID-19) pandemic.

## MATERIALS AND METHODS

### Data source and study population

The KNHANES is a cross-sectional survey of nationally representative samples of the civilian, non-institutionalized Korean population, conducted by the Korea Disease Control and Prevention Agency to evaluate health and nutritional status and to track significant chronic diseases [18,19]. This study utilized KNHANES data from 2011 to 2021 to examine the prevalence and trends of CKM syndrome. Participants aged 20 years or older with complete information on CKM syndrome component variables and SES were included in the analysis.

### Definition of chronic kidney disease syndrome

The 2023 AHA advisory defined CKM syndrome across 5 stages: stage 0 (no CKM risk factors), stage 1 (excess/dysfunctional adipose tissue), stage 2 (metabolic risk factors and CKD), stage 3 (subclinical CVD), and stage 4 (clinical CVD) [2]. Specific criteria for each stage, as defined by the AHA, are provided in Supplementary Material 1. Because data on subclinical CVD were not available, stages 3 and 4 were combined into a single category (stages 3-4) for analysis.

Table 1 shows the definition of CKM syndrome used in this study. Supplementary Material 2 provides the KNHANES variables and the definitions of CKM syndrome components.

Obesity was defined as a body mass index (BMI) of 25.0 kg/m^2^ rather than 23.0 kg/m^2^ as suggested by the AHA advisory for Asian populations [1]. Participants were considered to have hypertension if their systolic blood pressure was ≥ 140 mmHg, their diastolic blood pressure was ≥ 90 mmHg, if they had been diagnosed with hypertension, or if they were taking antihypertensive medications. Participants were classified as having diabetes if their fasting blood glucose level was ≥ 126 mg/dL, their glycated hemoglobin (HbA1c) was ≥ 6.5%, they had a history of diabetes diagnosis, or they were receiving treatment with diabetes-related medications or insulin. Individuals with total blood triglyceride levels ≥ 135 mg/dL were classified as having hypertriglyceridemia [19]. The estimated glomerular filtration rate (eGFR) was calculated using the Chronic Kidney Disease Epidemiology Collaboration equation (2021) [20], and CKD was reclassified using dipstick proteinuria values due to missing albuminuria data in KNHANES from 2015 to 2018 (Supplementary Material 3). Cases with an albumin-creatinine ratio (ACR) of less than 30 mg/g were replaced with “negative” or “trace” results on the dipstick test, and cases with an ACR of 30 mg/g or more were replaced with “positive” results. Individuals in the eGFR G3a category who were also positive for proteinuria presented classification challenges. To assess whether proteinuria could substitute for ACR, we calculated the kappa coefficient among participants with both ACR and dipstick proteinuria data (n= 33,915). A proteinuria result of +2 was defined as “moderate to high risk,” while a result of +3 was defined as “very high risk.” CVD was defined as a self-reported diagnosis of stroke, angina pectoris, or myocardial infarction (MI) [19].

### Statistical analysis

Weighted prevalence and 95% confidence intervals (CIs) were estimated. For trend analysis, the annual percent change (APC) was calculated using Joinpoint regression version 5.1.0 (National Cancer Institute, Rockville, MD, USA). Trends were considered significant when the p-value was < 0.05.

The overall analysis of CKM syndrome was stratified by gender and assessed across SES-related variables, including residential area (urban/rural, corresponding to *dong* vs. *eup* or *myeon* in KNHANES), education level (middle school or lower/high school/college or higher), and household income (low/lower middle/higher middle/high). Given the close relationship between SES and age, the association between SES and CKM syndrome was further examined by stratifying by age (20-49 and ≥ 50), and the proportions and trends of CKM syndrome stages were analyzed within each age group. Changes during the pandemic were assessed by comparing data from before the pandemic (2018-2019, n= 10,976) and during the pandemic (2020-2021, n= 9,935). Statistical significance for differences between groups was determined when the 95% CIs for each CKM stage prevalence did not overlap. Additional trend analysis was conducted after excluding the pandemic period (2020-2021) to examine its influence on CKM syndrome prevalence.

Additional analyses compared the distribution of age, gender, residential area, and education level between included and excluded participants and recalculated CKM stage using a BMI threshold of 23.0 kg/m^2^ for obesity. Analyses were performed using Stata/SE 18.0 (StataCorp., College Station, TX, USA) and SAS version 9.4 (SAS Institute Inc., Cary, NC, USA).

### Ethics statement

This study was approved by the Institutional Review Board (IRB) of Seoul National University Hospital (IRB No. E-2410-120-1578). Informed consent was waived by the IRB.

## RESULTS

**Figure 1.**
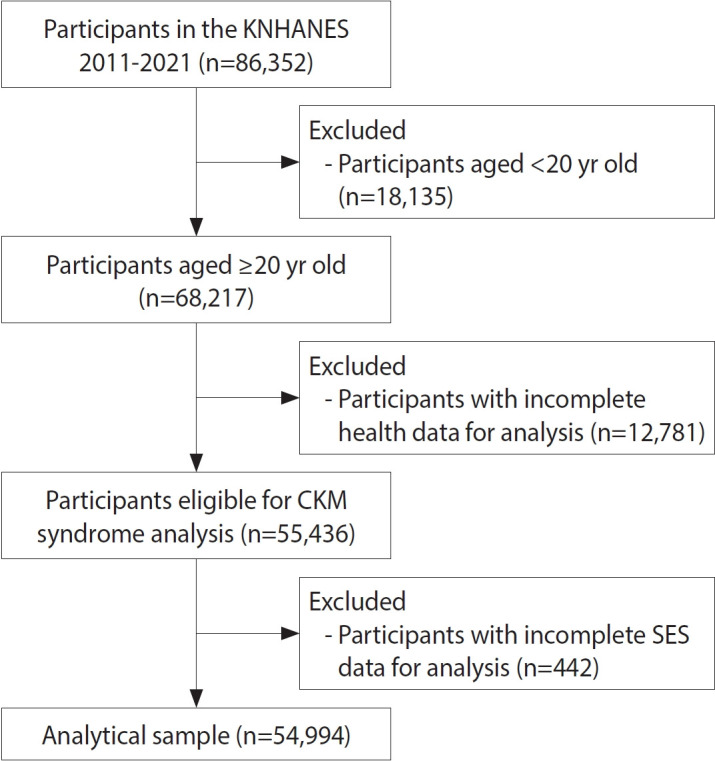
Flowchart of participation selection. KNHANES, Korean National Health and Nutrition Examination Survey; CKM, cardiovascular-kidney-metabolic; SES, socioeconomic status.

Among 86,352 participants (39,337 men and 47,015 women), we excluded individuals under 20 years old (n= 18,135), those with incomplete CKM component data (n= 12,781), and those with missing SES information (n= 442). This resulted in a final analytic sample of 54,994 adults (24,556 men and 30,438 women) (Figure 1).

The characteristics of the participants are shown in Table 2. There were no significant differences between included and excluded participants except for gender; the proportion of women was higher among those excluded (Supplementary Material 4).

### Chronic kidney disease classification kappa coefficient

The kappa test for the CKD definition among individuals with both ACR and proteinuria data showed moderate agreement (0.5213 for proteinuria 2+ and 0.5232 for proteinuria 3+), supporting the use of proteinuria-based CKD classification in place of the Kidney Disease Improving Global Outcomes (KDIGO) classification (Supplementary Material 5).

### The prevalence of cardiovascular-kidney-metabolic syndrome and components

**Figure 2.**
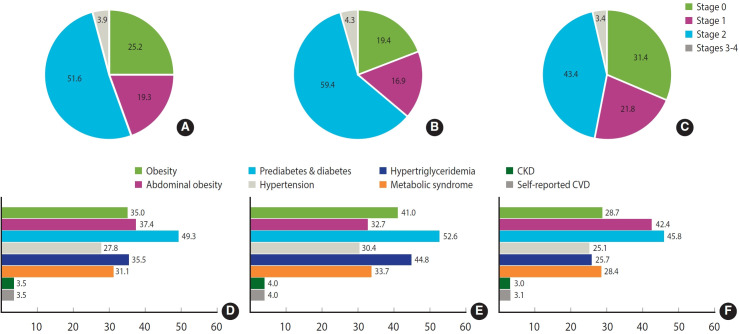
Cardiovascular-kidney-metabolic (CKM) syndrome and component prevalence stratified by gender. CKM syndrome stages in (A) all participants, (B) men, and (C) women. Each component of CKM syndrome in (D) all participants, (E) men, and (F) women. CKD, chronic kidney disease; CVD, cardiovascular disease.

Figure 2 and Table 2 display the prevalence of CKM syndrome and its components in Korean adults from 2011 to 2021. More than half of the participants were classified as CKM syndrome stage 2 (51.6%; 95% CI, 51.1 to 52.2). The prevalence of the other stages was as follows: stage 0 (25.2%; 95% CI, 24.7 to 25.8), stage 1 (19.3%; 95% CI, 18.9 to 19.7), and stages 3-4 (3.9%; 95% CI, 3.7 to 4.0). The prevalence of advanced CKM syndrome stages was higher in men (stage 2: 59.4%; 95% CI, 58.7 to 60.2; stages 3-4: 4.3%; 95% CI, 4.1 to 4.6) than in women (stage 2: 43.4%; 95% CI, 42.7 to 44.2; stages 3-4: 3.4%; 95% CI, 3.1 to 3.6). When stratified by age groups (20-49, 50-64, ≥ 65), older age groups had higher proportions of stages 3-4 and lower proportions of stage 0 (Supplementary Material 6).

With the exception of abdominal obesity, the prevalence of individual CKM syndrome components was higher in men than in women. When the overweight/obesity threshold was set at a BMI of 25.0 kg/m^2^, 5% of participants originally classified as stage 1 under the criteria of a BMI of 23.0 kg/m^2^ were reclassified as stage 0.

### Trends in cardiovascular-kidney-metabolic syndrome

**Figure 3.**
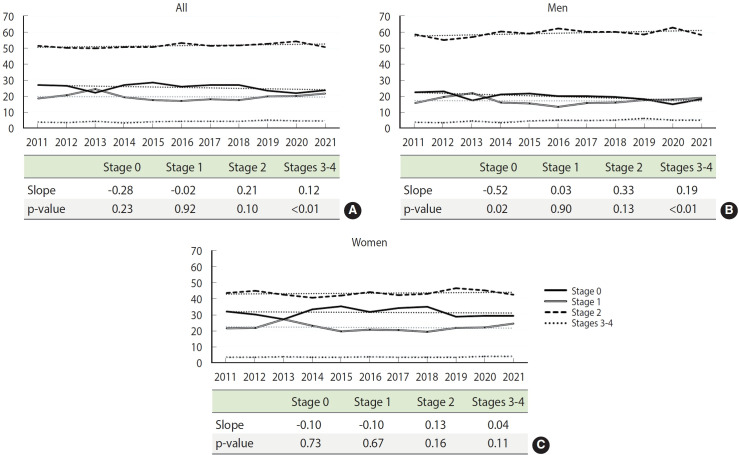
Trends in the prevalence of cardiovascular-kidney-metabolic (CKM) syndrome in (A) all participants, (B) men, and (C) women.

Figure 3 illustrates the annual trends in CKM syndrome prevalence. There was a significant increase in the proportion of stages 3-4 in the overall population (APC, 0.12; p< 0.01) and among men (APC, 0.19; p< 0.01). Additionally, a significant decrease in stage 0 was observed among men (APC, -0.52; p= 0.02). When the pandemic period was excluded (2011–2019), a significantly larger increase in the proportion of stages 3-4 was observed among all participants (APC, 3.65; p=0.01) and in men (APC, 6.28; p=0.01) (Supplementary Material 7). Supplementary Material 8 provides the annual prevalence rate of each CKM syndrome component and stage.

### Socioeconomic status and cardiovascular-kidney-metabolic syndrome

**Figure 4.**
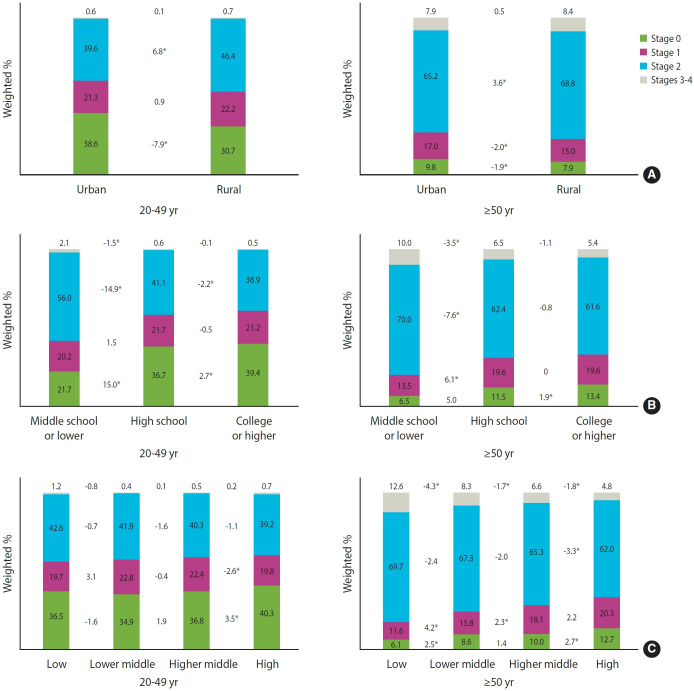
Socioeconomic status and prevalence of cardiovascular-kidney-metabolic (CKM) syndrome stratified by age group (before 50 years and after 50 years) by (A) residential area, (B) education level, and (C) household income. *p<0.05.

The overall weighted prevalence of CKM syndrome stratified by SES is presented in Figure 4 and Supplementary Material 9. The proportion of advanced CKM syndrome stages was higher in rural areas and among individuals with lower education levels in both age groups (20-49 and ≥ 50). Household income was also associated with CKM syndrome among participants aged 50 years and older, with higher income groups exhibiting a lower proportion of advanced CKM syndrome stages. This trend was similar when the analysis was stratified by gender.

### Pandemic and cardiovascular-kidney-metabolic syndrome

**Figure 5.**
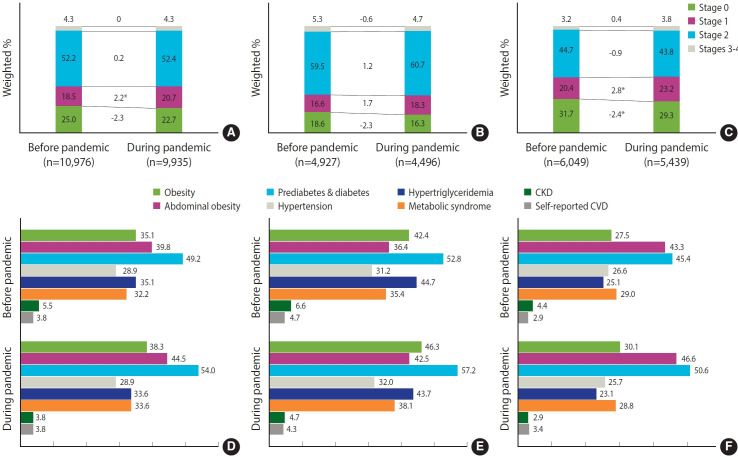
Comparison of overall cardiovascular-kidney-metabolic (CKM) syndrome prevalence before and during the pandemic in (A) all participants, (B) men, and (C) women. Prevalence of each CKM syndrome component in (D) all participants, (E) men, and (F) women. CKD, chronic kidney disease; CVD, cardiovascular disease. *p<0.05.

Figure 5 and Supplementary Material 10 present CKM syndrome prevalence and its components, stratified by gender and by pandemic period (before vs. during the pandemic). There was a significant increase in stage 1 during the pandemic, particularly among women. In addition, the prevalence of obesity, abdominal obesity, prediabetes, and diabetes increased significantly, whereas CKD prevalence decreased.

## DISCUSSION

This study examined the prevalence and trends of CKM syndrome in Korea using KNHANES data from 2011 to 2021, revealing that nearly three-quarters (74.8%) of Koreans are at risk. Trend analysis uncovered a marked increase in the prevalence of advanced CKM syndrome (stages 3-4) over the decade, with men being particularly affected. This surge mirrors the growing burden of cardiovascular and metabolic diseases in Korea [13,15-17]. Notably, men exhibited more severe CKM conditions than women, a disparity likely driven by a combination of genetic factors, lifestyle choices, behavioral patterns, and differences in health perception and healthcare-seeking [21-23].

Compared with data from the National Health and Nutrition Examination Survey (NHANES), Korea had lower proportions of individuals in advanced CKM syndrome stages: stage 0 (25.2% in Korea vs. 10.6% in the USA), stage 1 (19.3 vs. 25.9%), stage 2 (51.6 vs. 49.0%), and stages 3-4 (3.9 vs. 14.6%) [24]. The lower prevalence of advanced CKM stages in Korea may reflect differences in dietary habits, lifestyle, and healthcare systems between various Asian racial groups in the United States [25] and/or between Western and Asian countries [26-28]. Although the proportion of advanced CKM syndrome was lower in Korea than in the United States, trend analysis indicates that CKM syndrome is becoming a major health issue in Korea.

SES analysis revealed more advanced CKM syndrome among individuals in rural areas and those with lower education levels and household incomes. These findings are consistent with previous research showing that lower SES is associated with higher CKM risk factors and related-mortality [29-32]. In addition, our findings align with a previous United States study demonstrating an association between adverse socioeconomic conditions and higher CKM syndrome stages [33]. This disparity highlights the need for targeted public health interventions to address social determinants of health and improve access to healthcare and preventive services among disadvantaged populations.

The potential impact of the COVID-19 pandemic on CKM syndrome was also notable. The significant increase in stage 1 prevalence during the pandemic, particularly among women, suggests that lifestyle changes—such as reduced physical activity [34,35] and increased consumption of high-calorie foods [36,37]—may have exacerbated metabolic risk factors. The marked increase in stage 1 among women might be explained by a previous study that reported a significant decline in physical activity exclusively among women during the pandemic [35]. These findings underscore the importance of maintaining healthy behaviors during times of crisis and the need for public health strategies to mitigate the adverse health impacts of pandemics. However, data from 2020 to 2021 alone are insufficient to fully explore the pandemic’s impact. Continuous monitoring is necessary to determine whether this trend will persist or return to healthier stages. Furthermore, the gender differences in CKM syndrome prevalence before and during the pandemic warrant further research. For example, nutrition survey data from KNHANES could be used to analyze gender differences in dietary habits, offering insights into the observed trends, alongside longitudinal follow-up studies to track changes.

In this study, we set the BMI threshold for overweight/obesity at 25.0 kg/m^2^ rather than 23.0 kg/m^2^ as suggested by the AHA [1]. A report from the Asian Cohort Consortium suggests that BMI levels up to 27.5 kg/m^2^ may not significantly affect mortality [38]. When the BMI threshold was set at 23.0 kg/m^2^, the prevalence of overweight and/or obesity was 58.1%—23.1% higher than when using a threshold of 25.0 kg/m^2^. However, the lower BMI threshold resulted in only a 5% difference between stage 0 and stage 1 of CKM syndrome, likely because the CKM syndrome staging incorporates multiple factors such as abdominal obesity and diabetes.

Several limitations of this study should be acknowledged. First, distinguishing between stage 3 and stage 4 CKM syndrome was challenging due to the lack of subclinical CVD data in KNHANES. Second, the CVD outcomes available in KNHANES were limited to MI, angina, and stroke, potentially leading to an underestimation of advanced CKM syndrome prevalence. Although NHANES data include additional information on heart failure and heart attack, the differences between the 2 CVD definitions were minimal (data not shown). Third, the absence of ACR data in KNHANES from 2015 to 2018 necessitated the use of dipstick proteinuria values for CKD classification, which may be less precise than ACR measurements. Fourth, as this study is cross-sectional, it cannot establish causal relationships among the components of CKM syndrome. Prospective cohort studies are recommended to further explore the causal relationships, interactions, and relative importance of each CKM syndrome component. Nevertheless, our study is significant in that it defines CKM syndrome in an Asian population.

Future research should focus on longitudinal studies to better understand the progression of CKM syndrome and the effectiveness of targeted interventions. Additionally, improving data collection in national health surveys to include comprehensive measures of CVD and CKD will enhance the accuracy of CKM syndrome classification and facilitate more precise public health planning.

In conclusion, CKM syndrome represents a growing public health challenge in Korea with significant implications. This study provides guidance for developing policies aimed at reducing the burden of CKM syndrome by considering the interplay among cardiovascular, kidney, and metabolic diseases and socioeconomic factors.

## Figures and Tables

**Figure f6-epih-47-e2025005:**
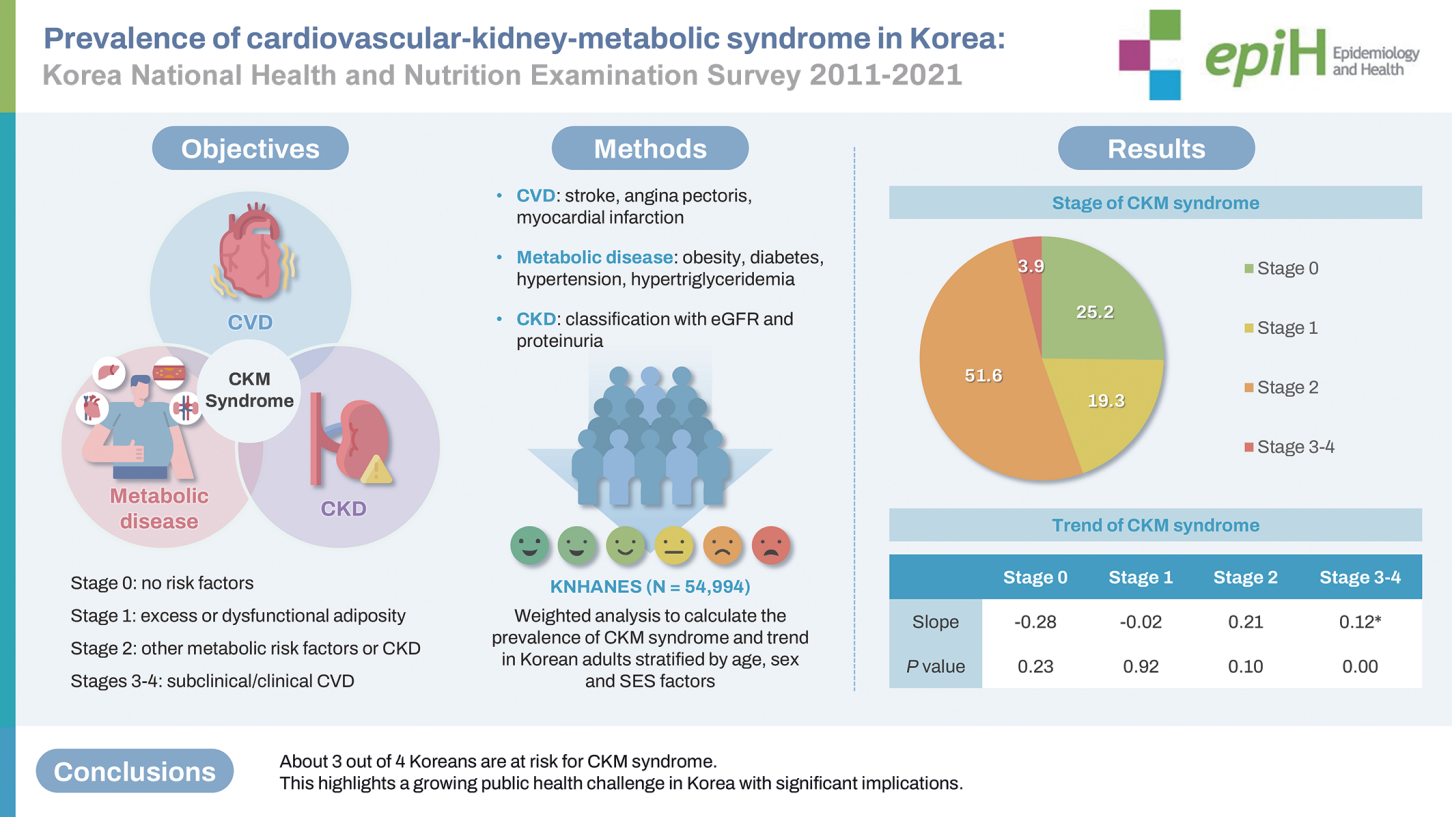


**Table 1.** Definition of CKM syndrome in this study

CKM syndrome stage	Definition
Stage 0	Normal BMI, WC, normoglycemia, normotension, normal lipid profile, no evidence of CKD, no subclinical and clinical CVD
No CKM risk factors	
Stage 1	BMI≥25.0 kg/m^2^ OR
Excess/dysfunctional adipose tissue	WC≥80/90 cm in women/men OR
	Prediabetes (FG: 100-125 mg/dL or HbA1c: 5.7-6.4%)
Stage 2	Hypertriglyceridemia (TG≥135 mg/dL) OR
Metabolic risk factors and CKD	Hypertension (SBP≥140 mmHg or DBP≥90 mmHg or self-reported diagnosis of hypertension or taking medicine) OR
	MetS^1^≥3 OR
	Diabetes (FG≥126 mg/dL or HbA1c≥6.5% or self-reported diagnosis of diabetes or taking medication or insulin) OR
	CKD (moderate to high risk)
Stage 3-4	Very high-risk CKD OR
Subclinical or clinical CVD in CKM syndrome	Clinical CVD (stroke, angina pectoris, myocardial infraction)

CKM, cardiovascular-kidney-metabolic; CKD, chronic kidney disease; CVD, cardiovascular disease; BMI, body mass index; WC, waist circumference; FG, fasting blood glucose; HbA1c, glycated hemoglobin; TG, total triglyceride; SBP, systolic blood pressure; DBP, diastolic blood pressure; MetS, metabolic syndrome.

1 MetS: (1) WC≥80/90 cm in women/men, (2) high-density lipoprotein cholesterol<40/50 mg/dL in men/women, (3) TG≥150 mg/dL, (4) blood pressure≥130/80 mmHg, or taking medication, (5) FG≥100 mm/dL.

**Table 2.** Characteristics of participants included in the analysis

Characteristics	Total (n=54,994, 100%)		Men (n=24,556, 51.2%)		Women (n=30,438, 48.8%)	
	Unweighted (n)	Weighted % (95% CI)	Unweighted (n)	Weighted % (95% CI)	Unweighted (n)	Weighted % (95% CI)
Age (yr)						
20-29	6,055	16.8 (16.3, 17.4)	2,987	18.5 (17.8, 19.3)	3,068	15.1 (14.5, 15.6)
30-39	8,525	18.4 (17.9, 19.0)	3,877	19.5 (18.8, 20.3)	4,648	17.3 (16.7, 18.0)
40-49	10,017	20.9 (20.3, 21.4)	4,481	21.3 (20.6, 22.0)	5,536	20.4 (19.8, 21.0)
50-59	11,118	20.6 (20.1, 21.1)	4,680	19.9 (19.3, 20.5)	6,438	21.3 (20.7, 21.9)
60-69	10,301	13.3 (13.0, 13.7)	4,586	12.7 (12.2, 13.1)	5,715	14.1 (13.6, 14.5)
70-79	7,261	7.9 (7.6, 8.2)	3,239	6.6 (6.3, 7.0)	4,022	9.2 (8.9, 9.6)
80-89	1,717	2.0 (1.9, 2.1)	706	1.4 (1.3, 1.6)	1,011	2.6 (2.4, 2.8)
Area of residence						
Dong	44,654	83.7 (82.1, 85.1)	19,773	83.3 (81.7, 84.8)	24,881	84.1 (82.6, 85.5)
*Eup/Myeon*	10,782	16.3 (14.9, 17.9)	4,964	16.7 (15.2, 18.3)	5,818	15.9 (14.5, 17.4)
Type of house						
General	26,603	49.2 (48.4, 50.0)	12,016	49.6 (48.6, 50.5)	14,587	48.8 (47.9, 49.7)
Apartment	28,833	50.8 (50.0, 51.6)	12,721	50.4 (49.5, 51.4)	16,112	51.2 (50.3, 52.1)
Individual income						
Low	13,229	24.5 (23.9, 25.2)	5,885	24.5 (23.7, 25.3)	7,344	24.6 (23.8, 25.3)
Lower middle	13,886	25.3 (24.7, 25.9)	6,197	25.4 (24.6, 26.2)	7,689	25.2 (24.6, 25.9)
Higher middle	13,954	25.2 (24.6, 25.8)	6,228	25.2 (24.5, 25.9)	7,726	25.1 (24.5, 25.8)
High	14,118	25.0 (24.2, 25.8)	6,318	24.9 (24.0, 25.8)	7,800	25.0 (24.2, 25.9)
Household income						
Low	10,164	14.4 (13.9, 15.0)	4,004	12.3 (11.7, 12.9)	6,160	16.6 (15.9, 17.3)
Lower middle	13,676	24.2 (23.6, 24.9)	6,034	23.6 (22.9, 24.4)	7,642	24.8 (24.1, 25.6)
Higher middle	15,153	29.6 (29.0, 30.3)	6,968	30.6 (29.8, 31.4)	8,185	28.7 (27.9, 29.4)
High	16,194	31.7 (30.8, 32.7)	7,622	33.5 (32.5, 34.5)	8,572	29.9 (28.9, 30.9)
Education level						
Middle school or lower	17,575	23.7 (23.0, 24.3)	6,169	17.8 (17.1, 18.4)	11,406	29.9 (29.0, 30.7)
High school	18,111	36.5 (35.9, 37.1)	8,720	38.8 (38.0, 39.6)	9,391	34.1 (33.3, 34.8)
College or higher	19,549	39.8 (39.0, 40.7)	9,774	43.4 (42.5, 44.4)	9,775	36.1 (35.2, 37.0)
BMI (kg/m^2^)						
Underweight (<18.5)	2,055	4.0 (3.8, 4.2)	531	2.4 (2.2, 2.7)	1,464	5.7 (5.3, 6.0)
Normal (18.5-22.9)	33,861	61.0 (60.5, 61.5)	14,148	56.6 (55.8, 57.3)	19,713	65.6 (64.9, 66.2)
Overweight/obesity (≥23.0)	19,078	35.0 (34.5, 35.5)	3,817	41.0 (40.2, 41.7)	9,261	28.7 (28.1, 29.4)
BMI (Asia criteria) (kg/m^2^)						
Underweight (<18.5)	2,055	4 (3.8, 4.2)	591	2.4 (2.2, 2.7)	1,464	5.7 (5.3, 6.0)
Normal (18.5-24.9)	20,875	37.9 (37.4, 38.4)	7,691	30.8 (30.1, 31.5)	13,184	45.4 (44.7, 46.1)
Overweight/obesity (≥25.0)	32,064	58.1 (57.6, 58.6)	16,274	66.8 (66.1, 67.5)	15,790	48.9 (48.2, 49.7)
WC (women/men) (cm)						
Normal (<80/90)	32,684	62.6 (62.0, 63.2)	16,309	67.3 (66.6, 68.1)	16,375	57.6 (56.8, 58.4)
Abdominal obesity (≥80/90)	22,310	37.4 (36.8, 38.0)	8,247	32.7 (31.9, 33.4)	14,063	42.4 (41.6, 43.2)
Glycemic status						
Normoglycemia	25,039	50.7 (50.1, 51.3)	10,176	47.4 (46.5, 48.2)	14,863	54.2 (53.4, 54.9)
Prediabetes	22,046	37.4 (36.9, 38.0)	10,321	39.5 (38.7, 40.3)	11,725	35.3 (34.6, 35.9)
Diabetes	7,909	11.9 (11.5, 12.2)	4,059	13.1 (12.7, 13.6)	3,850	10.6 (10.2, 11.0)
Hypertension						
No	36,724	72.2 (71.6, 72.7)	15,618	69.6 (68.9, 70.3)	21,106	74.9 (74.2, 75.5)
Yes	18,270	27.8 (27.3, 28.4)	8,938	30.4 (29.7, 31.1)	9,332	25.1 (24.5, 25.8)
Hypertriglyceridemia						
No	35,674	64.5 (64.0, 65.0)	13,685	55.2 (54.4, 55.9)	21,989	74.3 (73.7, 74.9)
Yes	19,320	35.5 (35.0, 36.0)	10,871	44.8 (44.1, 45.6)	8,449	25.7 (25.1, 26.3)
Metabolic syndrome						
No (<3)	36,160	68.9 (68.4, 69.4)	15,707	66.3 (65.6, 67.0)	20,453	71.6 (70.9, 72.2)
Yes (≥3)	18,834	31.1 (30.6, 31.6)	8,849	33.7 (33.0, 34.4)	9,985	28.4 (27.8, 29.1)
CKD (with ACR)^1^						
Low risk	30,639	92.1 (91.7, 92.4)	13,663	92.4 (91.8, 92.8)	16,976	91.8 (91.3, 92.3)
Moderate to high risk	3,082	7.5 (7.1, 7.9)	1,440	7.2 (6.8, 7.7)	1,642	7.8 (7.3, 8.3)
Very high risk	194	0.4 (0.4, 0.5)	106	0.4 (0.3, 0.5)	88	0.4 (0.3, 0.6)
CKD (with Upro 1^+^)^2^						
Low risk	52,606	96.5 (96.3, 96.7)	23,195	96 (95.7, 96.2)	29,411	97 (96.8, 97.2)
Moderate to high risk	2,203	3.3 (3.1, 3.4)	1,257	3.7 (3.5, 4.0)	946	2.8 (2.6, 3.0)
Very high risk	185	0.3 (0.2, 0.3)	104	0.3 (0.2, 0.4)	81	0.2 (0.2, 0.3)
CKD (with Upro 2^++^)^3^						
Low risk	52,606	96.5 (96.3, 96.7)	23,195	96 (95.7, 96.2)	29,411	97 (96.8, 97.2)
Moderate to high risk	2,110	3.1 (3.0, 3.3)	1,193	3.5 (3.3, 3.8)	917	2.7 (2.5, 2.9)
Very high risk	278	0.4 (0.3, 0.4)	168	0.5 (0.4, 0.6)	110	0.3 (0.2, 0.4)
Self-reported CVD						
No	52,319	96.5 (96.3, 96.6)	23,097	96 (95.8, 96.3)	29,222	96.9 (96.7, 97.1)
Yes	2,675	3.5 (3.4, 3.7)	1,459	4 (3.7, 4.2)	1,216	3.1 (2.9, 3.3)
CKM syndrome						
Stage 0	12,014	25.2 (24.7, 25.8)	3,904	19.4 (18.7, 20.0)	8,110	31.4 (30.7, 32.1)
Stage 1	10,309	19.3 (18.9, 19.7)	3,899	16.9 (16.3, 17.5)	6,410	21.8 (21.2, 22.4)
Stage 2	29,771	51.6 (51.1, 52.2)	15,160	59.4 (58.7, 60.2)	14,611	43.4 (42.7, 44.2)
Stage 3-4	2,900	3.9 (3.7, 4.0)	1,593	4.3 (4.1, 4.6)	1,307	3.4 (3.1, 3.6)
CKM syndrome (Asian BMI)						
Stage 0	9,755	20.2 (19.7, 20.7)	2,682	13.2 (12.7, 13.8)	7,073	27.5 (26.8, 28.2)
Stage 1	12,568	24.3 (23.9, 24.8)	5,121	23 (22.4, 23.7)	7,447	25.7 (25.1, 26.3)
Stage 2	29,771	51.6 (51.1, 52.2)	51,160	59.4 (58.7, 60.2)	14,611	43.4 (42.7, 44.2)
Stage 3-4	2,900	3.9 (3.7, 4.0)	1,593	4.3 (4.1, 4.6)	1,307	3.4 (3.1, 3.6)

CI, confidence interval; BMI, body mass index; WC, waist circumstance; CKD, chronic kidney disease; CVD, cardiovascular disease; CKM, cardiovascular-kidney-metabolic; eGFR, estimated glomerular filtration rate; ACR, albumin-to-creatinine ratio.

1 2015-2018 data excluded (total: 33,195, men: 15,209, women: 18,706).

2 Upro1^+^: individuals with an eGFR category of G3a who were positive for proteinuria were defined as “moderate to high risk.”

3 Upro2^++^: individuals with an eGFR category of G3a who were positive for proteinuria were defined as “very high risk.”

## Supplementary materials

Supplementary Material 1. Definition of CKM syndrome in AHA advisory

Supplementary Material 2. Definition of diseases components for CKM syndrome in KNHANES

Supplementary Material 3. CKD classification with proteinuria

Supplementary Material 4. Sensitive analysis between participants who were included and excluded

Supplementary Material 5. CKD classification using ACR and dipstick proteinuria test

Supplementary Material 6. Proportion of CKM syndrome stage strtified with age group and sex.

Supplementary Material 7. Trends in the prevalence of CKM syndrome in (A) all participants, (B) men, and (C) women from 2011 to 2019.

Supplementary Material 8. Characteristics of participants by year.

Supplementary Material 9. Socioeconomic status and prevalence of CKM syndrome stratified with before 50yr and after 50yr.

Supplementary Material 10. Comparison of each component of CKM syndrome and CKM syndrome stage before and during pandemic.
