# Supplementary material for: Prevalence of cardiovascular-kidney-metabolic syndrome in Korea: Korea National Health and Nutrition Examination Survey 2011-2021
Source: Epidemiol Health. 2025 Feb 14;47:e2025005. doi: 10.4178/epih.e2025005 (PMC12062855; doi:10.4178/epih.e2025005)
Supplement: Supplementary Material 1. — Definition of CKM syndrome in AHA advisory [file epih-47-e2025005-Supplementary-1.docx]

Supplementary Material 1. Definition of CKM syndrome in AHA advisory

| CKM syndrome stage | Definition |
| --- | --- |
| Stage 0: No CKM risk factors | normal BMI, waist circumference, normoglycemia, normotension, normal lipid profile,  no evidence of CKD, no subclinical and clinical CVD |
| Stage 1: Excess/dysfunctional adipose tissue | BMI ≥ 25kg/m^2^ (if, NHA ≥ 23kg/m^2^) OR WC ≥ 88/102 in women/men (if, NHA ≥ 80/90 in women/men) OR prediabetes (FG: 100-125mg/dL or HbA1c: 5.7-6.4%) |
| Stage 2: Metabolic risk factors and CKD | Hypertriglyceridemia (TG ≥ 135mg/dL) OR Hypertension (SBP ≥ 140mmHg or DBP ≥ 90mmHg or self-reported diagnosis of hypertension or taking medication) OR MetS^+^ ≥ 3 OR Diabetes (FG ≥ 126mg/dL or HbA1c ≥ 6.5% or self-reported diagnosis of diabetes or taking medication or insulin) OR CKD (moderate to high risk) |
| Stage 3: Subclinical CVD in CKM syndrome | Very high-risk CKD CKD stage G4 (eGFR: 15-29ml/min/1.73m^2^) OR  G5 (eGFR* < 15ml/min/1.73m^2^) OR  very high risk per KDIGO classification  [eGFR < 15ml/min/1.73m^2^ OR (eGFR: 15-29 ml/min/1.73m^2^ and ACR ≥ 300mg/g)] |
| Stage 4a: Clinical CVD in CKM syndrome without kidney failure | Clinical CVD (CHD, CHF, stroke, angina, heart attack or stroke) AND no kidney failure (eGFR ≥ 15ml/min/1.73m^2^) |
| Stage 4b: Clinical CVD in CKM syndrome with kidney failure | Clinical CVD (CHD, CHF, stroke, angina, heart attack or stroke) AND kidney failure (eGFR < 15ml/min/1.73m^2^) |

NHA, non-Hispanic Asian; CKD, chronic kidney disease; CVD, cardiovascular disease; FG, fasting blood glucose; TG, total triglyceride; SBP, systolic blood pressure; DBP, diastolic blood pressure; eGFR, estimated glomerular filtration rate; ACR, albumin-to-creatinine ratio; CHD, coronary heart disease; CHF, congestive heart failure;
Mets^+^(metabolic syndrome): (1) WC ≥ 88/102 in women/men (≥ 80/90 in non-Hispanic Asian), (2) HDL Cholesterol < 40/50 mg/dL in men/women, (3) TG ≥ 150 mg/dL, (4) BP ≥ 130/80 mmHg, or taking medication, (5) FG ≥ 100 mm/dL
