# Supplementary material for: Prevalence of cardiovascular-kidney-metabolic syndrome in Korea: Korea National Health and Nutrition Examination Survey 2011-2021
Source: Epidemiol Health. 2025 Feb 14;47:e2025005. doi: 10.4178/epih.e2025005 (PMC12062855; doi:10.4178/epih.e2025005)
Supplement: Supplementary Material 2. — Definition of diseases components for CKM syndrome in KNHANES [file epih-47-e2025005-Supplementary-2.docx]

Supplementary Material 2. Definition of diseases components for CKM syndrome in KNHANES

| Disease components | KNHANES variables | Categories | Definition |
| --- | --- | --- | --- |
| (1) BMI (kg/m^2^) | HE_BMI | Underweight | < 18.5 |
|  |  | Normal | 18.5 – 24.9 |
|  |  | Overweight/obesity | ≥ 25.0 |
| (2) WC (cm) | HE_wc | Normal | < 88/102 in women/men (if non-Hispanic Asian, < 80/90) |
|  |  | Abdominal obesity | ≥ 88/102 in women/men (if non-Hispanic Asian, ≥ 80/90) |
| (3) Glycemic status | HE_glu(FG), HE_HbA1c, DE1_dg(diagnosed Diabetes), DE1_31(insulin), DE1_32(medication) | Normoglycemia | FG < 100 mg/dL AND HbA1c < 5.7% |
|  |  | Prediabetes | FG 100 – 125 mg/dL OR HbA1c 5.7 – 6.4% |
|  |  | Diabetes | FG ≥ 126 mg/dL OR HbA1c ≥ 6.5% OR Diagnosed diabetes* |
|  |  | *Physician-diagnosed diabetes OR taking medication lowering blood sugar OR insulin | |
| (4) Hypertension | HE_sbp1~3, HE_dbp1~3, DI1_dg(diagnosed Hypertension), DI1_2(medication) | Normotension | BP < 140/90 mmHg |
|  |  | Hypertension | BP ≥ 140/90 mmHg OR use of antihypertensive medication |
| (5) Hypertriglyceridemia | HE_TG | Normal lipid profile | < 135 mg/dL |
|  |  | Hyperglyceridemia | ≥ 135 mg/dL |
| (6) Metabolic Syndrome | HE_wc, HE_HDL_st2, HE_TG, HE_sbp, HE_sbp, DI1_2, HE_glu | No MetS | < 3 of risk factors* |
|  |  | MetS | ≥ 3 of risk factors |
|  |  | *MetS risk factors includes the following criteria: (1) WC ≥ 80/90 in women/men, (2) HDL cholesterol < 40/50 in men/women, (3) TG ≥ 150, (4) SBP ≥ 130 or DBP ≥ 80 or use of antihypertensive medication, (5) FG ≥ 100 or taking medication | |
| (7) CKD with Upro2 | HE_CREA, HE_Upro | Low risk (no CKD) | eGFR ≥ 60 mL/min AND Proteinuria negative, trace |
|  |  | Moderate to high risk | eGFR ≥ 60 mL/min AND Proteinuria positive OR eGFR 30-60 mL/min AND Proteinuria negative, trace |
|  |  | Very high risk | eGFR < 30 mL/min OR eGFR 30-60 mL/min AND Proteinuria positive |
| (8) CVD | DI3_dg(stroke), DI4_dg(CHD) | No CVD | No self-reported CVD |
|  |  | CVD | stroke, angina pectoris, myocardial infraction |
