# Supplementary material for: Prevalence of cardiovascular-kidney-metabolic syndrome in Korea: Korea National Health and Nutrition Examination Survey 2011-2021
Source: Epidemiol Health. 2025 Feb 14;47:e2025005. doi: 10.4178/epih.e2025005 (PMC12062855; doi:10.4178/epih.e2025005)
Supplement: Supplementary Material 3. — CKD classification with proteinuria [file epih-47-e2025005-Supplementary-3.docx]

Supplementary Material 3. CKD classification with proteinuria

|  |  | Proteinuria | | |
| --- | --- | --- | --- | --- |
| eGFR categories | | Negative, Trace (±) | Positive | |
| G1 | ≥90 | 1 | 2 | |
| G2 | 60-89 | 1 | 2 | |
| G3a | 45-59 | 2 | 2 | 3 |
| G3b | 30-44 | 2 | 3 | |
| G4 | 15-29 | 3 | 3 | |
| G5 | <15 | 3 | 3 | |

1: low risk (No CKD or at the lowest risk for CKD getting worse), 2: Moderate to high risk (increased risk for CKD getting worse), 3: Very high risk (at the highest risk for CKD getting worse
