# Supplementary material for: Prevalence of cardiovascular-kidney-metabolic syndrome in Korea: Korea National Health and Nutrition Examination Survey 2011-2021
Source: Epidemiol Health. 2025 Feb 14;47:e2025005. doi: 10.4178/epih.e2025005 (PMC12062855; doi:10.4178/epih.e2025005)
Supplement: Supplementary Material 4. — Sensitive analysis between participants who were included and excluded [file epih-47-e2025005-Supplementary-4.docx]

Supplementary Material 4. Sensitive analysis between participants who were included and excluded

|  |  | Included participants (N = 54,994) | | Excluded participants (N = 12,781) | |
| --- | --- | --- | --- | --- | --- |
|  |  | Weighted % | 95% CI | Weighted % | 95% CI |
| Age | |  |  |  |  |
|  | 20-39 | 35.2 | 34.6 – 36.0 | 36.2 | 34.9 – 37.6 |
|  | 40-59 | 41.5 | 40.9 – 42.1 | 34.7 | 33.4 – 35.9 |
|  | 60+ | 23.3 | 22.7 – 23.9 | 29.1 | 27.9 – 30.3 |
| Sex | |  |  |  |  |
|  | Men | 51.2 | 50.8 – 51.6 | 40.8 | 39.6 – 41.9 |
|  | Women | 48.8 | 48.4 – 49.2 | 59.2 | 58.1 – 60.4 |
| Residential area | |  |  |  |  |
|  | Urban | 83.7 | 82.1 – 85.1 | 80.4 | 78.4 – 82.5 |
|  | Rural | 16.3 | 14.9 – 17.9 | 19.6 | 17.5 – 21.6 |
| Education level | |  |  |  |  |
|  | Middle school or lower | 23.7 | 23.0 – 24.3 | 30.0 | 28.4 – 31.5 |
|  | High school | 36.5 | 35.9 – 37.1 | 34.8 | 33.2 – 36.4 |
|  | College or higher | 39.8 | 30.8 – 32.7 | 35.2 | 33.5 – 36.9 |
