# Supplementary material for: Prevalence of cardiovascular-kidney-metabolic syndrome in Korea: Korea National Health and Nutrition Examination Survey 2011-2021
Source: Epidemiol Health. 2025 Feb 14;47:e2025005. doi: 10.4178/epih.e2025005 (PMC12062855; doi:10.4178/epih.e2025005)
Supplement: Supplementary Material 5. — CKD classification using ACR and dipstick proteinuria test [file epih-47-e2025005-Supplementary-5.docx]

Supplementary Material 5. CKD classification using ACR and dipstick proteinuria test

|  |  | CKD with Upro1 | | | CKD with Upro2 | | |
| --- | --- | --- | --- | --- | --- | --- | --- |
|  |  | Low risk | Moderate to high risk | Very high risk | Low risk | Moderate to high risk | Very high risk |
| CKD  With  ACR | Low risk | 30,517 | 122 | 0 | 30,517 | 122 | 0 |
|  | Moderate to high risk | 1,934 | 1,146 | 2 | 1,934 | 1,121 | 27 |
|  | Very high risk | 0 | 84 | 110 | 0 | 53 | 141 |
|  | Kappa value | 0.5213 | | | 0.5232 | | |
