# Supplementary material for: Prevalence of cardiovascular-kidney-metabolic syndrome in Korea: Korea National Health and Nutrition Examination Survey 2011-2021
Source: Epidemiol Health. 2025 Feb 14;47:e2025005. doi: 10.4178/epih.e2025005 (PMC12062855; doi:10.4178/epih.e2025005)
Supplement: Supplementary Material 7. — Trends in the prevalence of CKM syndrome in (A) all participants, (B) men, and (C) women from 2011 to 2019. [file epih-47-e2025005-Supplementary-7.docx]

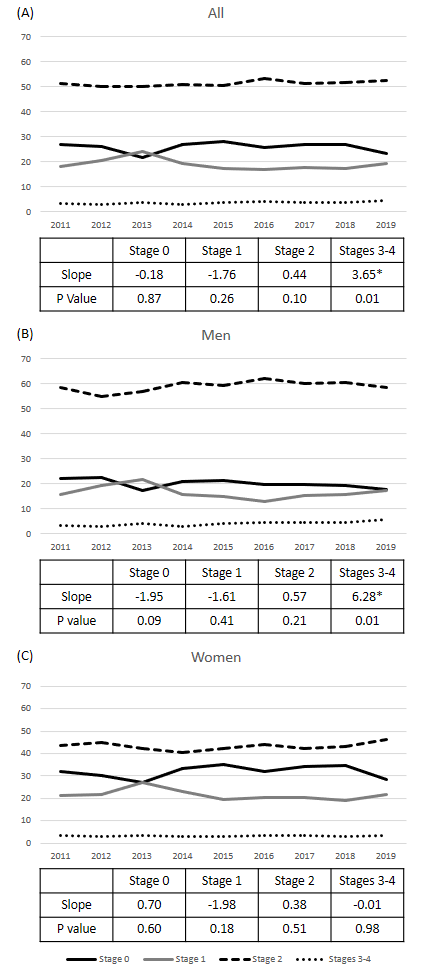


Supplementary Material 7. Trends in the prevalence of CKM syndrome in (A) all participants, (B) men, and (C) women from 2011 to 2019.
* P value < 0.05
